# CD19-CAR-T Cells Bearing a KIR/PD-1-Based Inhibitory CAR Eradicate CD19^+^HLA-C1^−^ Malignant B Cells While Sparing CD19^+^HLA-C1^+^ Healthy B Cells

**DOI:** 10.3390/cancers12092612

**Published:** 2020-09-13

**Authors:** Lei Tao, Muhammad Asad Farooq, Yaoxin Gao, Li Zhang, Congyi Niu, Iqra Ajmal, Ying Zhou, Cong He, Guixia Zhao, Jie Yao, Mingyao Liu, Wenzheng Jiang

**Affiliations:** Shanghai Key Laboratory of Regulatory Biology, School of Life Sciences, East China Normal University, Shanghai 200241, China; 52161300026@stu.ecnu.edu.cn (L.T.); 52181300056@stu.ecnu.edu.cn (M.A.F.); 52181300027@stu.ecnu.edu.cn (Y.G.); 52171300027@stu.ecnu.edu.cn (L.Z.); 51171300098@stu.ecnu.edu.cn (C.N.); 52171300059@stu.ecnu.edu.cn (I.A.); 51181300130@stu.ecnu.edu.cn (Y.Z.); 52161300027@stu.ecnu.edu.cn (C.H.); 52191300040@stu.ecnu.edu.cn (G.Z.); 52201300031@stu.ecnu.edu.cn (J.Y.)

**Keywords:** CD19-CAR-T, B cell aplasia, KIR, PD-1, inhibitory CAR

## Abstract

**Simple Summary:**

CD19-targeted chimeric antigen receptor (CAR) T (CD19-CAR-T) cell therapy usually causes B cell aplasia because of “on-target off-tumor” toxicity. The aim of the study was to assess the concept that the introduction of an inhibitory CAR (iCAR) into CAR-T cells could alleviate the side effect of CD19-CAR-T cell therapy. The results showed that CD19-CAR-T cells with a novel KIR (killer inhibitory receptor) /PD-1 (programmed death receptor-1)-based inhibitory CAR (iKP-19-CAR-T) exhibited more naïve, less exhausted phenotypes and preserved a higher proportion of central memory T cells (T_CM_). Furthermore, iKP-19-CAR-T cells exerted the similar level of cytotoxicity on CD19^+^HLA-C1^−^ Burkitt’s lymphoma cells compared to CD19-CAR-T cells while sparing CD19^+^HLA-C1^+^ healthy human B cells both in vitro and in the xenograft model. Our data demonstrates that the KIR/PD-1-based inhibitory CAR can be a promising strategy to avoid B cell aplasia caused by CD19-CAR-T cell therapy.

**Abstract:**

B cell aplasia caused by “on-target off-tumor” toxicity is one of the clinical side effects during CD19-targeted chimeric antigen receptor (CAR) T (CD19-CAR-T) cells treatment for B cell malignancies. Persistent B cell aplasia was observed in all patients with sustained remission, which increased the patients’ risk of infection. Some patients even died due to infection. To overcome this challenge, the concept of incorporating an inhibitory CAR (iCAR) into CAR-T cells was introduced to constrain the T cells response once an “on-target off-tumor” event occurred. In this study, we engineered a novel KIR/PD-1-based inhibitory CAR (iKP CAR) by fusing the extracellular domain of killer cell immunoglobulin-like receptors (KIR) 2DL2 (KIR2DL2) and the intracellular domain of PD-1. We also confirmed that iKP CAR could inhibit the CD19 CAR activation signal via the PD-1 domain and CD19-CAR-T cells bearing an iKP CAR (iKP-19-CAR-T) exerted robust cytotoxicity in vitro and antitumor activity in the xenograft model of CD19^+^HLA-C1^−^ Burkitt’s lymphoma parallel to CD19-CAR-T cells, whilst sparing CD19^+^HLA-C1^+^ healthy human B cells both in vitro and in the xenograft model. Meanwhile, iKP-19-CAR-T cells exhibited more naïve, less exhausted phenotypes and preserved a higher proportion of central memory T cells (T_CM_). Our data demonstrates that the KIR/PD-1-based inhibitory CAR can be a promising strategy for preventing B cell aplasia induced by CD19-CAR-T cell therapy.

## 1. Introduction

CD19-CAR-T (CD19-targeted chimeric antigen receptor T-) cells are the first cell therapy products for the treatment of relapsed or refractory B cell acute lymphoblastic leukemia (B-ALL) that were approved by US Food and Drug Administration (FDA) in 2017 [1,2]. Since then, CD19-CAR-T has brought a gigantic revolution in the field of immunotherapy because of the high percentage rate of complete remission (CR) in several blood-related malignancies [3,4,5,6]. While some challenges increase the risk of treatment failures, such as an “on-target off-tumor” adverse event instigating B cell aplasia, i.e., CD19-CAR-T cells kill all healthy B cells because of CD19 expressed in all B cells [7,8,9]. B cell aplasia contributes to hypogammaglobulinemia, which is one of the main factors leading to infection in patients [10]. Although these patients were administrated an intravenous immunoglobulin to maintain IgG levels, concomitant bacterial, viral, and fungal infections were still observed [11,12].

At present, some strategies have been developed to overcome the “on-target off-tumor” effect. Since the ideal specific target is almost non-existent in reality, it is a good idea to target structurally differentiated proteins. For example, the engineered CAR-T cells targeting the integrin β7 activated conformation was specifically effective against multiple myeloma (MM) without damaging normal hematopoietic cells [13]. Similarly, recognition of the Tn-glyco form of MUC1 by engineering CAR-T cells exhibited target-specific cytotoxicity to cellular adenocarcinoma [14]. According to another strategy, splitting 4-1BB domain and CD3ζ domain and fusing them together with two different single chain fragment variable regions (scFv) respectively, T cells would be entirely active if only two antigens were recognized at the same time [15]. Likewise, the same result was found using the “And gate” approach, in which logical control of CAR-T cells responses needed two different antigen engagements [16,17,18]. Although these strategies can reduce the incidence of the “on-target off-tumor” effect, they are not universal and not easy to implement.

In contrast to the strategies outlined earlier, inhibitory CAR (iCAR) is a versatile and implementable solution to subdue the “on-target off-tumor” effect by providing a negative signal to regulate T cell activation. In 2013, Fedorov et al. developed a PD-1-based iCAR strategy and they provided a proof of concept that CAR-T cells expressing an iCAR could discriminate between off target cells and target cells and functioned in a temporary and reversible manner [19].

KIRs are the most important inhibitory receptors expressed predominantly in NK cells and a small subset of T cells [20]. They can dampen the activation of NK cells after interacting with the human leukocyte antigen (HLA) ligands expressed on the surface of normal cells [21,22]. Tumor cells downregulate HLA to escape from the T cells immune surveillance [23,24,25]. Data from the Human Protein Atlas Database demonstrate that HLA-C is low or non-expressed on most tumor cell lines, but highly or moderately expressed in normal tissues. PD-1 is an inhibitory protein expressed in activated T cells to limit the excessive activation of T cells [26,27,28]. In this study, we engineered a novel iCAR consisting of the extracellular domain of KIR2DL2, CD8a hinge and transmembrane, and the intracellular domain of PD-1. This KIR/PD-1-based iCAR was termed as iKP CAR. We speculated that when KIR2DL2 recognized HLA-C1 on normal cells, iKP CAR would deliver a negative signal to inhibit T cells response via the PD-1 domain, meanwhile, iKP CAR did not work in the absence of HLA-C1 on tumor cells, so that iKP CAR could discriminate between normal cells (HLA-C1^+^) and tumor cells (HLA-C1^−^). Simultaneously, we hoped that CD19-CAR-T cells with an iKP CAR could eliminate CD19^+^HLA-C1^−^ malignant B cells, while reducing the damage to CD19^+^HLA-C1^+^ healthy B cells.

## 2. Results

### 2.1. iKP CAR Doesn’t Affect CAR Expression, Viability, Proliferation and Subsets of CD19-CAR-T Cells

**Figure 1:**
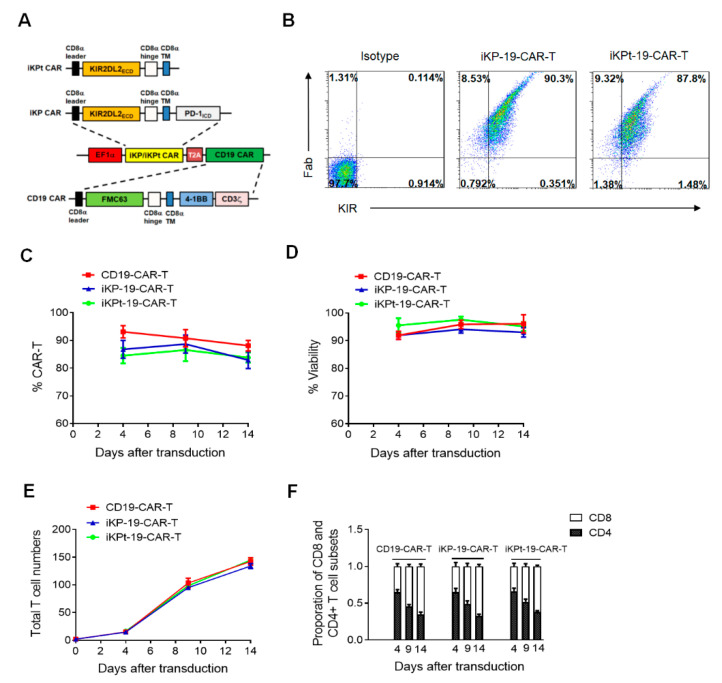
iKP CAR construction and expression. (**A**) Schematic diagram of the bicistronic vector expressing iKP/iKPt CAR and CD19 CAR. (**B**–**F**) iKP-19-CAR-T/iKPt-19-CAR-T cells or CD19-CAR-T cells were cultured for 14 days in X-VIVO media supplemented with 100U/mL IL-2. Representative iKP/iKPt CAR and CD19 CAR expression in iKP-19-CAR-T/iKPt-19-CAR-T cells were detected on day 4 by flow cytometry using PE-anti-human KIR antibody and Alexa Flour 647-anti-mouse F(ab’)2 antibody (scFv of CD19 CAR is from mouse) (*n* = 4 different donors) (**B**). Detection of CD19 CAR-positive rate in iKP-19-CAR-T/iKPt-19-CAR-T and CD19-CAR-T on day 4, day 9 and day 14 by flow cytometry (*n* = 4 different donors) (**C**). Viability (**D**) or total cell numbers (**E**) of iKP-19-CAR-T/iKPt-19-CAR-T cells and CD19-CAR-T cells were also measured on day 4, day 9 and day 14 using Beckman Coulter counter (*n* = 4 different donors). Proportion of CD8+ and CD4+ T cell subsets in iKP-19-CAR-T/iKPt-19-CAR-T cells or CD19-CAR-T cells on day 4, day 9 and day 14 was measured using APC-anti-human CD8 antibody and PerCP-anti-human CD4 antibody (*n* = 4 different donors) (**F**). Three experiments were performed using PBMCs from each donor. Error bars represent ± SD.

We designed iKP CAR by fusion of the extracellular domain of KIR2DL2 and the intracellular domain of PD-1 with CD8a hinge and transmembrane. Whereas, an iKP CAR with truncated PD-1 domain (named as iKPt CAR) was used as a negative control (upper panel of Figure 1A). Next, the commercially synthesized iKP/iKPt CAR was cloned into a vector expressing a CD19 CAR in which both CARs were separated by a T2A sequence (lower panel of Figure 1A). The bicistronic vector expressing CD19 CAR and iKP/iKPt CAR was used to package a lentivirus and transduced in Human Primary T cells from healthy donors to produce iKP-19-CAR-T/iKPt-19-CAR-T cells. Flow cytometry analysis showed that iKP-19-CAR-T/iKPt-19-CAR-T cells expressed analogous amounts of both CD19 CAR and iKP/iKPt CAR (Figure 1B), which ensured that iKP-19-CAR-T/iKPt-19-CAR-T cells would receive activation and suppression signals evenly. To study whether iKP/iKPt CAR affected characteristics of CD19-CAR-T cells, CD19-CAR-T and iKP-19-CAR-T/iKPt-19-CAR-T cells were cultured in X-VIVO media supplemented with 100 U/mL IL-2 for 14 days and the cells were analyzed by flow cytometry at different time points. We found that both CD19-CAR-T and iKP-19-CAR-T/iKPt-19-CAR-T cells displayed a similar expression level of CD19 CAR (Figure 1C), cell viability (Figure 1D), cell proliferation (Figure 1E) and proportion of CD8+ and CD4+ cells (Figure 1F). These results indicated that iKP CAR had no impact on CAR expression, viability, proliferation and subsets of CD19-CAR-T cells.

### 2.2. iKP CAR Functions via PD-1 Signaling Upon Interacting with HLA-C1

**Figure 2:**
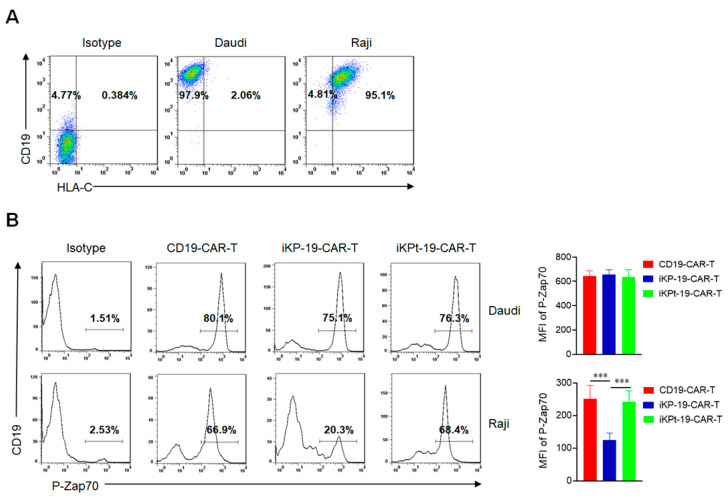
Dephosphorylating P-Zap70 by iKP CAR via intracellular PD-1 domain. (**A**) Flow cytometric analysis of CD19 and HLA-C1 expression in Daudi cells or Raji cells by using APC-anti-human CD19 and PE-anti-human HLA-C antibodies. (**B**) Expression analysis of P-Zap70 in different CAR-T cells by flow cytometry. iKP-19-CAR-T/iKPt-19-CAR-T cells and CD19-CAR-T cells were exposed to Daudi cells or Raji cells for 6 h at a 1:1 ratio in RPMI-1640 medium, stained with PE-anti-human P-Zap70 antibody and MFI of P-Zap70 was statistically analyzed (*n* = 4 different donors). All the experiments were conducted in triplicate manner using PBMCs from each donor. *** *p* < 0.001. Error bars represent ± SD. The CD19 CAR positive rate was unified using UT cells in all the co-culture experiments in this study.

To investigate whether iKP CAR could regulate the CD19 CAR signal through the intracellular PD-1 domain once it interacted with HLA-C1, Daudi cells (CD19^+^HLA-C1^−^) and Raji cells (CD19^+^HLA-C1^+^) were used as target cells and the presence of CD19 and HLA-C1 was analyzed by flow cytometry (Figure 2A). Next, CD19-CAR-T cells, iKP-19-CAR-T cells and iKPt-19-CAR-T cells were exposed to Daudi cells or Raji cells in RMPI-1640 medium after the CAR positive rate was unified. It was reported that PD-1 recruited SHP2 to dephosphorylate P-Zap70 to inhibit T cell activation [29,30]. In current study, the phosphorylated Zap70 (P-Zap70) was determined by flow cytometry six hours later. The results showed that the expression level of P-Zap70 in CD19-CAR-T cells, iKP-19-CAR-T cells, or iKPt-19-CAR-T cells was similar (Figure 2B) when exposed to Daudi cells, while the expression level of P-Zap70 in iKP-19-CAR-T cells was remarkably decreased compared to CD19-CAR-T cells or iKPt-19-CAR-T cells (Figure 2B) when exposed to Raji cells. The data indicated that in the absence of HLA-C1 (Daudi cells), iKP CAR would not affect the activation signal of CD19 CAR, however in the presence of HLA-C1 (Raji cells), iKP CAR would dephosphorylate P-Zap70 via intracellular PD-1 domain. Regardless of the presence of HLA-C1, iKPt CAR had no effect on the CD19 CAR activation signal, therefore we only compared the functional differences between iKP-19-CAR-T cells and CD19-CAR-T cells in further experiments.

### 2.3. iKP CAR Renders CD19-CAR-T Cells in Less Differentiated and Less Exhausted State Prior to Antigen Engagement

**Figure 3:**
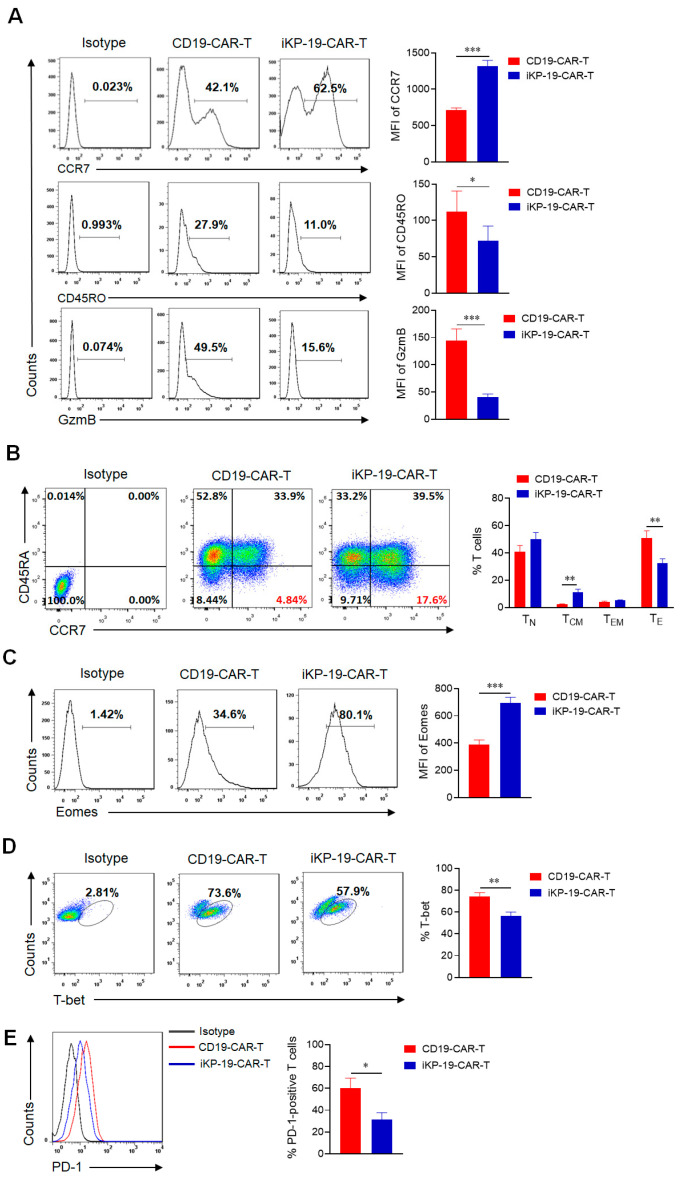
Characteristics of iKP-19-CAR-T cells and CD19-CAR-T cells. iKP-19-CAR-T cells or CD19-CAR-T cells were cultured for 10 days in X-VIVO media supplemented with 100 U/mL IL-2. (**A**) The expression of T cell differentiation markers in CAR-T cells was analyzed by flow cytometry using PE/Cy7-anti-human CCR7 antibody, PE-anti-human-CD45RO antibody and PE-anti-human GzmB antibody (*n* = 4 different donors). (**B**) The frequency of naïve (T_N_; CCR7^+^CD45RA^+^), T_CM_ (CCR7^+^CD45RA^−^), effector memory (T_EM_; CCR7^−^CD45RA^−^) or effector (T_E_; CCR7^−^CD45RA^+^) T cells were analyzed by flow cytometry using PE/Cy7-anti-human CCR7 antibody and FITC-anti-human CD45RA antibody (*n* = 4 different donors). (**C**) The expression of transcription factor Eomes in CAR-T cells was analyzed by flow cytometry using FITC-anti-human Eomes antibody (*n* = 4 different donors). (**D**) The expression of transcription factor T-bet in CAR-T cells was analyzed by flow cytometry using PE-anti-human T-bet antibody (*n* = 4 different donors). (**E**) The expression of T cell exhaustion marker PD-1 in CAR-T cells was analyzed by flow cytometry using FITC-anti-human PD-1 antibody (*n* = 4 different donors). All experiments were performed in triplicate manner using PBMCs from each donor and MFI or percentage was statistically analyzed. * *p* < 0.05, ** *p* < 0.01, *** *p* < 0.001. Error bars represent ± SD.

IL-2 activates T cells through PI3K-Akt-mTOR and MAPK signaling pathways [31,32], but high concentration of IL-2 in the media will cause excessive activation of T cells. PD-1 plays an opposite role to IL-2 also through PI3K-Akt-mTOR and MAPK signaling pathways to inhibit T cells activation and proliferation [33,34,35]. Since donor T cells expressed HLA-C1 as well (Supplementary Materials Figure S1), the interaction between iKP CAR and HLA-C1 on T cells could provide a negative PD-1 signal to suppress IL-2 induced T cells activation, which probably affected the properties of iKP-19-CAR-T cells different from CD19-CAR-T cells. Firstly, the differentiation status of CAR-T cells prior to antigen exposure was evaluated. Flow cytometry analysis for CCR7, CD45RO, and GzmB was performed in CD19-CAR-T cells and iKP-19-CAR-T cells. We found an increased CCR7 expression, decreased CD45RO and GzmB expression in iKP-19-CAR-T cells compared to CD19-CAR-T cells (Figure 3A). These results suggested that iKP-19-CAR-T cells were in a less differentiated state than CD19-CAR-T cells [35]. Furthermore, we observed that the percentage of T_CM_ (CD45RA^−^CCR7^+^) in iKP-19-CAR-T cells was higher than that in CD19-CAR-T cells (Figure 3B). Due to the high expression of Eomes and low expression of T-bet dedicated to T_CM_ [36,37], we analyzed these two transcription factors by flow cytometry and the results showed that iKP-19-CAR-T cells had a higher Eomes expression level and a lower T-bet expression level compared to CD19-CAR-T cells (Figure 3C,D). In addition, PD-1 expression in iKP-19-CAR-T cells was lower than that found in CD19-CAR-T cells (Figure 3E), while TIM-3 and LAG-3 expression showed no significant changes (data not shown). Therefore, our results demonstrated that the integration of iKP CAR into CD19-CAR-T cells leads to less differentiated and less exhausted T cell phenotypes.

Although the KIR-PD-1 signal possibly attenuated the IL-2 signal, iKP CAR did not impair the cell proliferative capacity of CD19-CAR-T cells (Figure 1E), which meant that the T cells still had sufficient signal for proliferation. To investigate this, the expression level of P-Zap70 in iKP-19-CAR-T cells and CD19-CAR-T cells was analyzed by using flow cytometry. We found that there was no difference in the expression level of P-Zap70 between these two CAR-T cells (Supplementary Materials Figure S2).

### 2.4. CD19-CAR-T Cells Bearing an iKP CAR Eradicate CD19^+^HLA-C1^−^ Daudi Cells and Present Lower Cytotoxicity on CD19^+^HLA-C1^+^ Normal B Cells In Vitro

**Figure 4:**
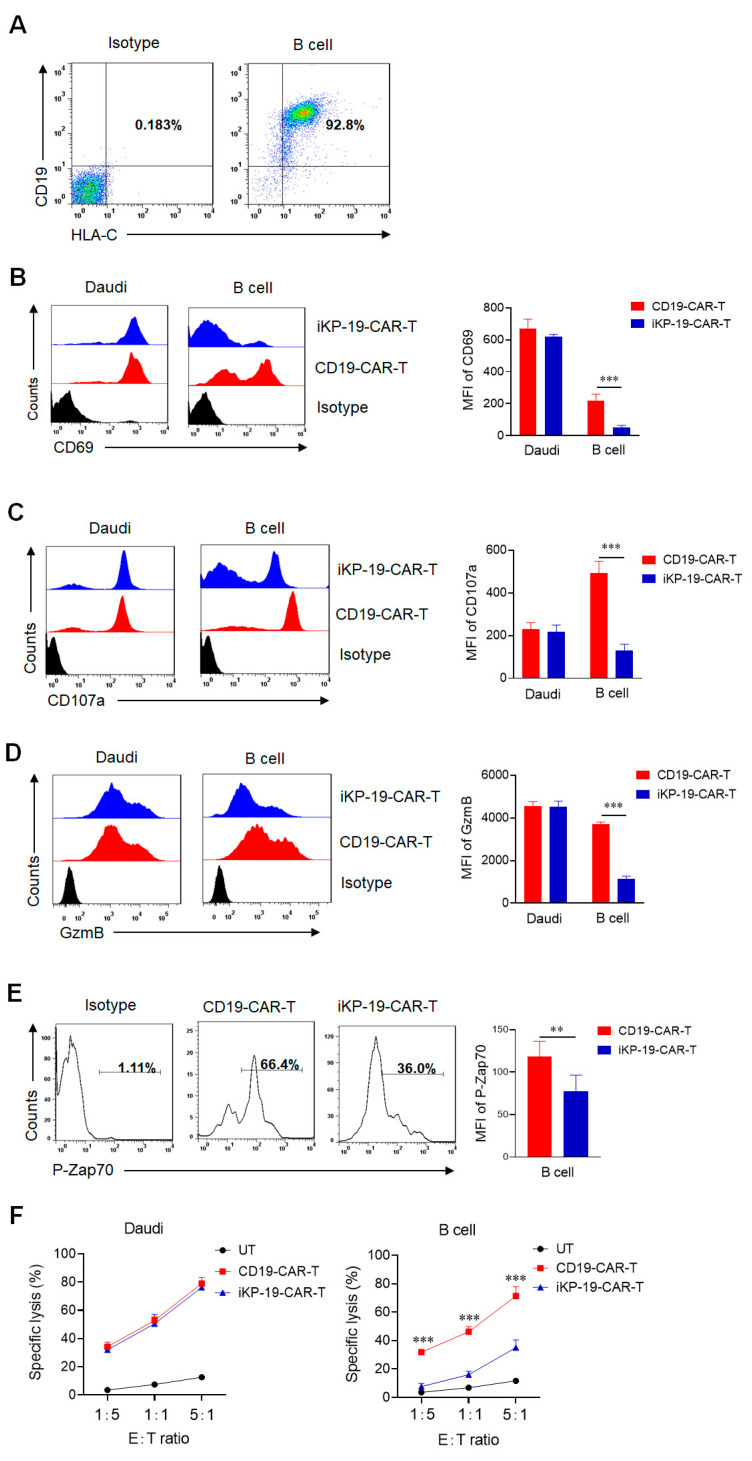
In vitro cytotoxicity of iKP-19-CAR-T cells against CD19^+^HLA-C1^−^ Daudi cells or CD19^+^HLA-C1^+^ normal B cells. (**A**) Representative CD19 and HLA-C1 expression in B cells. (**B**–**E**) After CD19 CAR rate was unified, iKP-19-CAR-T cells and CD19-CAR-T cells were co-cultured with Daudi cells or normal B cells at a 1:1 ratio for 6 h in RPMI-1640 medium. The expressions of activation marker CD69 (**B**), degranulation marker CD107a (**C**), GzmB (**D**), and signal molecule P-Zap70 (**E**) in CAR-T cells was detected by flow cytometry using PE-anti-human CD69 antibody, PE-anti-human CD107a antibody, PE-anti-human GzmB antibody and PE-anti-human P-Zap-70 antibody. MFI was statistically measured from three independent experiments (*n* = 4 different donors). (**F**) LDH assay was performed to test the cytotoxicity of iKP-19-CAR-T cells and CD19-CAR-T cells against Daudi cells or normal B cells after 6h co-culture at different E:T ratio (*n* = 4 different donors). All experiments were performed in triplicate manner using PBMCs of every donor. ** *p* < 0.01, *** *p* < 0.001. Error bars represent ± SD.

In order to study whether iKP-19-CAR-T cells could distinguish between malignant B cells and normal B cells in vitro, CD19^+^HLA-C1^+^ normal B cells from healthy donors were identified as one of the target cells (Figure 4A). Next iKP-19-CAR-T cells and CD19-CAR-T cells were co-cultured with Daudi cells or healthy B cells in RMPI-1640 medium for 6 h at a 1:1 ratio, respectively. T cell activation was assessed by using flow cytometry. The results revealed that the expression level of the activation marker CD69, the degranulation marker CD107a, and GzmB was similar in both iKP-19-CAR-T cells and CD19-CAR-T cells when co-culturing with Daudi cells. However, a significantly lower level of these molecules in iKP-19-CAR-T cells was observed when co-culturing with normal B cells (Figure 4B–D). Moreover, a lower level of P-Zap70 was observed in iKP-19-CAR-T cells compared to CD19-CAR-T cells when exposed to normal B cells (Figure 4E), but it had a similar expression level in both CAR-T cells when exposed to Daudi cells (Figure 2B). The data indicated that iKP CAR could constrain the activation of CD19-CAR-T cells effectively upon engagement of HLA-C1 on normal B cells.

To evaluate the cytotoxicity of iKP-19-CAR-T cells or CD19-CAR-T cells against different target cells, an LDH release assay was executed. At a different E:T ratio, iKP-19-CAR-T cells showed the same strong cytotoxicity on Daudi cells as CD19-CAR-T cells, a killing rate of almost 80% was observed at a 5:1 ratio (Figure 4F). However, the cytotoxicity of iKP-19-CAR-T cells on normal B cells was decreased dramatically compared to CD19-CAR-T cells, iKP CAR reduced the cytotoxicity by 51% at a 5:1 ratio, and the reduction effect was more pronounced at a lower E:T ratio (Figure 4F). The results suggested that the combination of iKP CAR and CD19 CAR could reduce the damage of CD19-CAR-T cells on CD19^+^HLA-C1^+^ normal B cells without decreasing the cytotoxicity on CD19^+^HLA-C1^−^ malignant B cells in vitro.

### 2.5. CD19-CAR-T Cells Bearing an iKP CAR Release Less Cytokines and Express Lower Exhaustion Markers during Lysing Malignant B Cells

**Figure 5:**
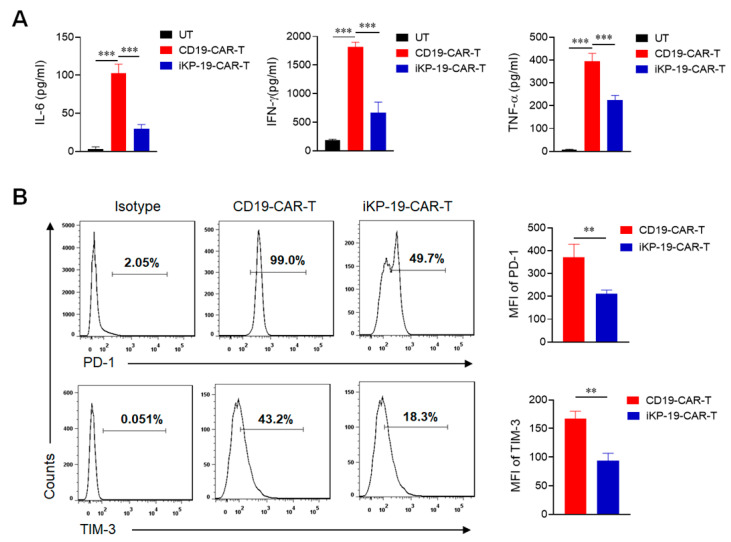
Cytokines release and exhaustion marker expression of iKP-19-CAR-T cells after coculture with Burkitt’s lymphoma Daudi cells. iKP-19-CAR-T cells or CD19-CAR-T cells and Daudi cells were co-cultured in 96 well plate at a 1:1 ratio for 6 h in RPMI complete media. (**A**) Cell culture media were collected and IL-6, IFN-γ and TNF-α were determined by flow cytometry using CBA assay kit (*n* = 4 different donors). (**B**) CAR-T cells were collected and stained with FITC-anti-human PD-1 antibody and PE/Cy7-anti-human TIM-3 antibody to measure the expression of exhaustion markers PD-1 and TIM-3 (*n* = 4 different donors). MFI was statistically analyzed from three different experiments of each donor. ** *p* < 0.01, *** *p* < 0.001. Error bars represent ± SD.

Further, we measured the cytokines in media where CAR-T cells were cocultured with Daudi cells at a 1:1 ratio. The data showed that iKP-19-CAR-T cells released lower levels of cytokines including IL-6, IFN-γ and TNF-α compared to CD19-CAR-T cells (Figure 5A), which was beneficial to prevent cytokine release syndrome (CRS) [38,39]. Next, we tested the expression of surface markers on iKP-19-CAR-T cells or CD19-CAR-T cells and found that iKP-19-CAR-T cells expressed lower exhaustion markers of PD-1 and TIM-3 than CD19-CAR-T cells (Figure 5B). This data proved that CD19-CAR-T cells with an iKP CAR might have better properties than CD19-CAR-T cells.

### 2.6. CD19-CAR-T Cells Bearing an iKP CAR Discern CD19^+^HLA-C1^−^ Burkitt’s Lymphoma Cell Line and CD19^+^HLA-C1^+^ Healthy B Cells In Vivo

**Figure 6:**
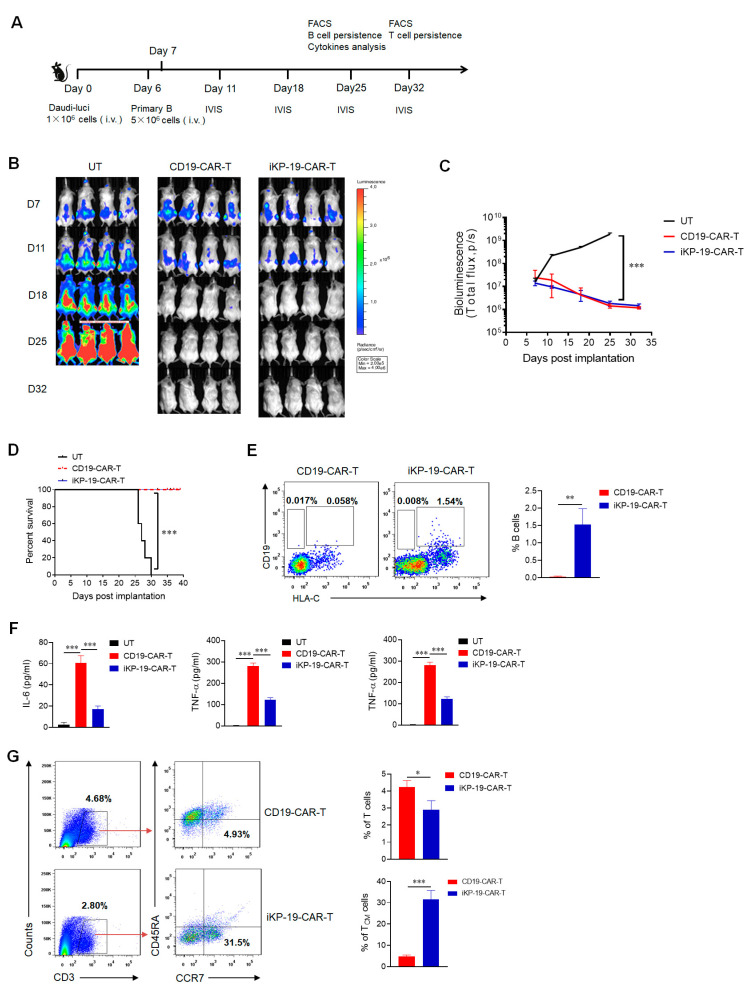
Controlling B cell malignancy effectively with sparing normal B cells in vivo of iKP-19-CAR-T cells. (**A**) Schematic representation of in-vivo experimental design. NCG mice (*n* = 4/group) were i.v. injected with 1 × 10^6^ luciferase-expressed Daudi cells (Daudi-luc) on day 0. After 6 days, 5 × 10^6^ normal B cells were administered. At day 7, 5 × 10^6^ UT cells, CD19-CAR-T cells and iKP-19-CAR-T cells were i.v. injected. IVIS imaging was performed to monitor tumor burden at day 11, 18, 25, 32. At day 25, normal B cells and cytokines in PB were analyzed. At day 32, T cells persistence was evaluated. (**B**) Representative bioluminescence images of Daudi-luc cells-derived tumor growth in the xenograft model. (**C**) Bioluminescence kinetics of Daudi-luc cells-derived tumor growth in the xenograft model. (**D**) Kaplan-Meier survival curve of mice. (**E**) Representative presence of Daui cells (CD19^+^HLA-C1^−^) or normal B cells (CD19^+^HLA-C1^+^) in mice at day 25 was determined by flow cytometry using APC-anti-human CD19 antibody and PE-anti-human HLA-C antibody. (**F**) Cytokine secretion of IL-6, IFN-γ and TNF-α in PB at day25 was measured by using CBA assay kit. (**G**) Flow cytometer analysis of total numbers of T cells and central memory T cells (T_CM_) in different group of xenograft mice. T cells (CD3^+^) or T_CM_ (CCR7^+^CD45RA^−^) were detected from PB by using APC-anti CD3 antibody, FITC-anti-human CD45RA antibody and PE/Cy7-anti-human CCR7 antibody. All the experiments were performed with 4 mice per group. * *p* < 0.05, ** *p* < 0.05, *** *p* < 0.001. Error bars represent ± SD.

To study the cytotoxicity of iKP-19-CAR-T cells on CD19^+^HLA-C1^−^ Burkitt’s lymphoma cells or CD19^+^HLA-C1^+^ normal B cells in vivo, Daudi cells expressing luciferase were generated and were injected intravenously (i.v.) into six-week-old NOD-Prkdc^em26Cd52^IL2rg^em26Cd22^/Nju (NCG) mice via tail veins on day 0 and normal B cells were injected in the same way on day 6 (Figure 6A). The mice were divided into three separate groups (*n* = 4), and each group of mice received untransduced T cells (UT), CD19-CAR-T cells, or iKP-19-CAR-T cells intravenously. As shown in the IVIS imaging system, both iKP-19-CAR-T cells and CD19-CAR-T cells controlled B cell malignancy effectively as compared to UT cells (Figure 6B). When compared to the mice of the UT group, the total bioluminescence of tumors in mice of the iKP-19-CAR-T group or CD19-CAR-T group was decreased to a significantly lower level (Figure 6C). A 100% survival rate was recorded in those mice who received an iKP-19-CAR-T cell or CD19-CAR-T cell treatment on day 39, but all mice in the UT group died on day 30 (Figure 6D). On day 25, peripheral blood (PB) from mouse orbit was collected to analyze the persistence of normal B cells and cytokines release, respectively. Neither CD19^+^HLA-C1^−^ Daudi cells nor CD19^+^HLA-C1^+^ normal B cells were detected in the CD19-CAR-T group (Figure 6E). However, certain quantities of normal B cells (1.54%) still existed but Daudi cells were not detected in the iKP-CD19-CAR-T group (Figure 6E). The results demonstrated that iKP-19-CAR-T cells could eliminate malignant B cells while still sparing normal B cells in vivo. More importantly, compared with CD19-CAR-T-treated mice, less cytokines such as IL-6, IFN-γband TNF-α in the sera from iKP-19-CAR-T-treated mice were detected (Figure 6F), which implied that iKP-19-CAR-T cells were safer than CD19-CAR-T cells. Furthermore, on day 32, we found that the mice treated with CD19-CAR-T cells had more T cell survival than the mice treated with iKP-19-CAR-T cells, but among the surviving T cells, the iKP-19-CAR-T-treated mice had a higher percentage of T_CM_ or less-differentiated cells (Figure 6G), which suggested that iKP-19-CAR-T cells would provide longer-term antitumor activities.

## 3. Discussion

CARs use scFv structure to recognize target antigens on cancer cells. The safety of CAR-T cells depends on the specificity of target antigens. However, most antigens are also expressed in normal cells, hence the “on-target off-tumor” effect is inevitable. This behavior of CAR-T cells causes severe side effects in the body systems expressing the target antigens [3,10,11,40]. Current clinical solutions are to use high-dose corticosteroid for treatment when “on-target off-tumor” events occur [41]. This immunosuppressive drug controls off-target toxicity at the cost of abolishing the T cells antitumor effect.

Fedorov et al. provided a model to elucidate the possibility to apply iCAR to regulate the function of CAR-T cells [19]. As we know, NK cells can discriminate between normal cells and abnormal cells that do not express adequate amounts of HLA such as cancer cells, virus-infected cells, etc [42]. Whether they are activated depends on the integrated signal of positive and negative signals. KIR is one of the important inhibitory receptors and exerts an inhibitory function to constrain NK cell response upon recognizing HLA on normal cells [43]. Based on the activation mechanism of NK cells, we fused the PD-1 intracellular signal domain and the extracellular recognition domain of KIR2DL2 to develop an iKP/PD-1-based iCAR (iKP CAR), and the data demonstrated that T cells co-expressing CD19 CAR and iKP CAR could discern between malignant B cells and normal B cells in vitro and in vivo.

Two factors are important for iCAR design. Firstly, the target of iCAR should be widely expressed on normal tissue cells, but rarely expressed on cancer cells. Obviously, HLA is an ideal target for iCAR. As HLA-C1 subtype has a high expression frequency in humans [44], we choose the extracellular domain of KIR2DL2 (whose ligand is HLA-C1) as the recognition domain of iCAR. Secondly, the intracellular signal domain should respond quickly and strongly in a transiently reversible manner once the extracellular recognition domain binds to the target. PD-1 is a powerful inhibitory molecule in T cells that dephosphorylates the TCR signal in a few hours after interacting with PD-L1 [45]. The dephosphorylation event is a dynamic and reversible process, which can ensure that T cell activity can restore during engagement of target cells. Therefore, we used the intracellular domain of PD-1 as the signaling domain of iCAR in the current study.

In our study, some characteristics of CD19-CAR-T cells were not affected by iKP CAR. Data showed that iKP-19-CAR-T cells had similar CAR transduction efficacy, cell viability, proliferation and CD8/CD4 ratio to CD19-CAR-T cells in X-VIVO media supplemented with IL-2. However, some characteristics were different. Prior to antigen engagement, iKP-19-CAR-T cells displayed lower differentiated (increased CCR7, decreased CD45RO and GzmB expression), less exhausted (lower PD-1 expression) phenotypes, and had an elevated proportion of T_CM_ or less-differentiated cells. The reason might be that the negative signaling of iKP CAR suppressed the IL-2 signal and regulated the related gene expression (up-regulated Eomes expression and down-regulated T-bet expression), thereby inhibiting T cell differentiation and exhaustion simultaneously. A higher percentage of T_CM_ or less differentiated cells in the peripheral blood from the mice treated with iKP-19-CAR-T cells had also been observed in vivo. Retrospective analysis from published CAR-T cell clinical studies had revealed that an elevated proportion of T_CM_ or less differentiated CAR-T cells provided superior antitumor efficacy [36]. Both in vitro and in vivo, we found that HLA-C1 on T cells did not reduce the toxicity of iKP-19-CAR-T cells on Daudi cells. Therefore, we speculated that iKP-19-CAR-T cells obtained an activation pattern similar to NK cells due to iCAR functioning in a temporary and reversible manner [19].

Subsequently, we demonstrated that the novel iKP CAR here had an ability to discern malignant B cells and normal B cells both in vitro and in vivo. Compared to CD19-CAR-T cells, iKP-19-CAR-T cells had an equivalent level of T cell activation, degranulation, and cytotoxic potential against Daudi cells, while all these were reduced significantly against normal B cells in vitro. Furthermore, we found that B cell malignancy could be controlled effectively by both CAR-T cells, but normal B cells were still detectable in the xenograft mice model treated with iKP-19-CAR-T cells while they could not be found in the mice treated with CD19-CAR-T cells. In contrast to CD19-CAR-T cells, the amount of CRS-associated cytokines IL-6, TNF-α and IFN-γ of iKP-19-CAR-T cells was decreased notably during lysing Daudi cells in vitro and this phenomenon was also observed in vivo, which indicated that iKP-19-CAR-T cells were safer than CD19-CAR-T cells. The result was similar to a CD19-CAR-T cells variant reported by Ying et al. [46]. This was possibly because iKP-19-CAR-T cells were not always activated, their activation was suppressed by the negative signaling provided by HLA-C1 expressed in normal B cells or T cells themselves. Therefore, the “missing self” activation mechanism like NK cells confers to iKP-19-CAR-T cells to control malignant B cells effectively and spare normal B cells.

“On-target off-tumor” toxicity has seriously limited the clinical application of CAR-T cells in the treatment of solid tumors. It has been reported that HER2-targeted CAR-T cell treatment for colon cancer caused a patient death because of CAR-T cells off target to pulmonary tissues [47]. In theory, iKP CAR can also reduce the “on-target off-tumor” toxicity of HER2-CAR-T cells to normal tissue cells. So far, our lab has detected the efficiency of iKP CAR in HER2-CAR-T cells therapy in vitro and in vivo, and similar results have been acquired (data not published).

**Figure 7:**
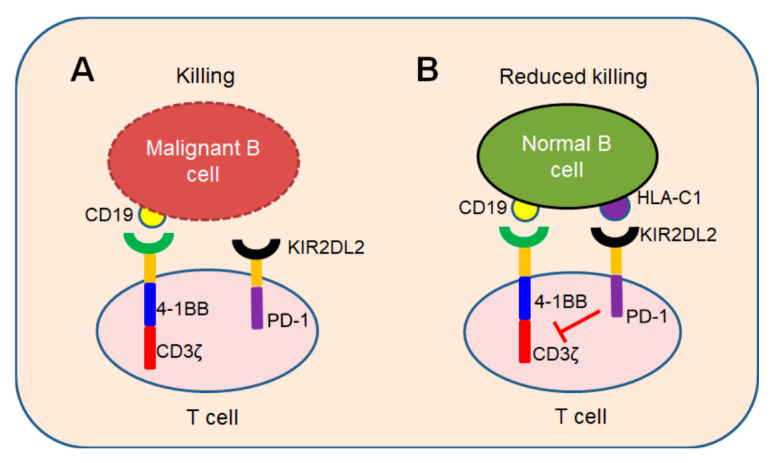
“Missing self” mechanism of iKP CAR. T cells co-expressing CD19 CAR and iKP CAR exploit “missing self” activation mechanism similar to NK cells. (**A**) T cells are activated by CD19 CAR and kill malignant B cells upon recognizing CD19 on malignant B cell tumors. (**B**) CD19 CAR activation signal is inhibited by PD-1 signal when iKP CAR is engaged to “self-HLA-C1” on normal B cells and iKP-19-CAR-T cells do not kill normal B cells.

In conclusion, we demonstrated a novel iKP CAR can recognize HLA-C1 and deliver an inhibitory signaling to T cells. T cells can be activated by CD19 CAR and kill malignant B cells because iKP CAR does not work (Figure 7A). Once it recognizes “self-HLA-C1” on normal B cells, iKP CAR will deliver an inhibitory signaling via the intracellular PD-1 domain to halt T cell activation mediated by CD19 CAR (Figure 7B). This “missing self” activation mechanism like NK cells confers to iKP-19-CAR-T to control malignant B cells effectively and spare normal B cells in vitro and in vivo. The effectiveness of iKP CAR in the human body needs to be verified in future clinical trials.

## 4. Materials and Methods

### 4.1. Cell Lines

293T cell line was preserved in our lab and propagated in Dulbecco modified eagle medium (DMEM) (Invitrogen, Carlsbad, CA, USA) supplemented with 10% heat-inactivated fetal bovine serum (FBS) (Thermo Fisher Scientific, Waltham, MA, USA) and 1% penicillin/streptomycin (P/S) (Thermo Fisher Scientific, Waltham, MA, USA). The Burkitt’s lymphoma Daudi cells and Raji cells were purchased from ATCC (Manassas, VA, USA) and maintained in RPIM-1640 medium (Invitrogen, Carlsbad, CA, USA) supplemented with 10% FBS and 1% P/S. Daudi cell line expressing Luciferase (Daudi-luc) was generated by stably transducing fire-fly luciferase in wild-type Daudi cells.

### 4.2. T-Cell and B-Cell Isolation

Human Peripheral blood was used to isolate peripheral blood mononuclear cells (PBMC) by density centrifugation method according to the manufacturer’s guideline (Sigma, San Louis, MO, USA). Primary T cells were positively selected using a mixture of (1:1) anti-CD4 and anti-CD8 microbeads (Miltenyi, Koln, Germany) and normal B cells were negative selected by using B Cell Isolation Kit II (Miltenyi) from PBMCs according to the manufacturer’s protocol. Both of them were cultured in X-VIVO^TM^ 15 serum free medium (LONZA, Basel, Switzerland) supplemented with 1% P/S, and stored in liquid nitrogen. All fresh blood was collected under a protocol approved by the Ethics Committee of East China Normal University (m20190315), following written informed consent.

### 4.3. iKP CAR/iKPt CAR Construction

iKP CAR was generated by linking the nucleotide sequence of extracellular domain of KIR2DL2 (AA22-245, Uniprot sequence ID P43627.) and intracellular domain of PD-1 (AA192-288, Uniprot sequence ID Q15116) with CD8a hinge and transmembrane nucleotide sequence. iKPt CAR was used as a negative control designed by truncating PD-1 domain of iKP CAR. Then, the commercially synthesized iKP CAR or iKPt CAR was cloned into a pCDH lentiviral vector expressing a CD19 CAR separated by a T2A sequence (GAGGGCAGAGGAAGTCTTCTAACATGCGGTGACGTGGAGGAGAATCCCGGCCCT).

### 4.4. Lentiviral Vector Production

Lentiviral supernatant was produced in the 293T packaging cell line according to the routine protocol [48]. In brief, 70% confluent 10 cm cell culture plates of 293T cells were co-transfected with 5 μg pCDH vector plasmid, 5 μg psPAX2 (Gag/pol/REV) and 3 μg pMD2.G (VSVG envelope) packaging plasmid using Lipofectamine 2000 transfecting reagent (Thermo Fisher Scientific, Waltham, MA, USA). Medium was replaced after 12-h transfection. The 48-h and 72-h viral supernatants were collected, combined and ultra-centrifuged at 25,000 rpm for 2 h to obtain concentrated lentivirus and stored at −80 °C for future use.

### 4.5. iKP-19-CAR-T/iKPt-19-CAR-T Cell Manufacture

After T cells were thawed and stimulated with CD3/CD28 Dynabeads (Miltenyi, Bergisch Gladbach, Germany) for 2 days, T cells were transduced with lentiviral vectors mentioned above at a MOI of 20, and maintained at 1 × 10^6^ cells per ml in X-VIVO media with 100 U/mL of human IL-2 (Peprotech, Rocky Hill, NJ, USA) as described [44,49]. iKP-19-CAR-T/iKPt-19-CAR-T cells expansion was carried out for 14 days. Absolute cell counts and viability were obtained with a Coulter Counter (Beckman Coulter, Brea, CA, USA). The expression of CARs, T cell differentiation markers CCR7, CD45RO and GzmB, phonotype markers CCR7 and CD45RA, transcription factors Eomes and T-bet, exhaustion markers PD-1, TIM-3 and LAG-3, signal molecule P-Zap70 and CD8^+^/CD4^+^ T cells ratio were analyzed using flow cytometry.

### 4.6. Analysis of iKP CAR Function

Multiple sets of co-cultivation experiments were performed to study iKP CAR function. In every experiment, CD19 CAR-positive rate was unified using untransduced T cells (UT) and target cells were seeded at a density of 10^4^ cells per well in 96 well plate in a triplicate manner. To verify whether iKP CAR performed PD-1 function after recognizing HLA-C1, CD19-CAR-T cells and iKP-19-CAR-T/iKPt-19-CAR-T cells were co-cultured with Daudi cells (CD19^+^HLA-C1^−^) or Raji cells (CD19^+^HLA-C1^+^) at 1:1 effector cells to target cells ratio (E:T) for 6 h, then the expression of P-Zap70 in different CAR-T cells was assayed by flow cytometry; In order to further investigate whether iKP CAR could suppress the activation of T cells, iKP-19-CAR-T cells and CD19-CAR-T cells were co-cultured with Daudi cells or normal B cells (CD19^+^HLA-C1^+^) at a 1:1 ratio for 6 h, then the expression level of CD69, CD107a, GzmB and P-Zap70 was determined by using flow cytometry.

### 4.7. Flow Cytometry

CD19-CAR-T cells or iKP-19-CAR-T cells were gated by CD19 CAR+, then the expression of the related molecules in CAR-T cells was analyzed. Especially, transcription factors T-bet and Eomes staining was performed using the FoxP3 TF Staining Buffer Set (eBioscience, San Diego, CA, USA) and intracellular proteins GzmB and P-Zap70 staining was performed using Fixation and Permeabilization Solution Kit (BD Biosciences, San Jose, CA, USA) according to the manufacturer’s instruction respectively. In brief, 2 × 10^6^ T cells or co-cultured cells were collected and washed twice with PBS, then cells were resuspended with 1 mL 1 × TF FIX/Perm Buffer per tube (or 250 μL BD 1 × Fixation/Permeabilization solution per tube) for 45 min at 4 °C to lyse nuclear membranes (or cell membranes). The supernatants were removed after centrifuging and cells were washed with 1 mL 1 × Perm/wash Buffer per tube (or 1 mL 1 × BD Perm/Wash Buffer per tube) twice. Next, cells were incubated with respective antibodies for 30 min at 4 °C and washed with PBS twice, protein expression levels were tested by flow cytometry. The following fluorescently-labeled monoclonal antibodies (mAbs) were used in this study: APC-anti-human CD19 (#555415, BD Biosciences, San Jose, CA, USA), PE-anti-human HLA-C (#566372, BD Biosciences, San Jose, CA, USA), PE-anti-human KIR (#556071, BD Biosciences, San Jose, CA, USA), PE-anti-human CD45RO (#561889, BD Biosciences, San Jose, CA, USA), PE-anti-human CD107a (#555801, BD Biosciences, San Jose, CA, USA), Alexa Flour 647-anti- mouse F(ab’)2 antibody (#115-605-006, Jackson ImmunoResearch, West Grove, PA, USA), PerCP-anti-human CD4 (#317431, BioLegend, San Diego, CA, USA), APC-anti-human CD8 (#344722, BioLegend, San Diego, CA, USA), FITC-anti-human PD-1 (#621612, BioLegend, San Diego, CA, USA), PE/Cy7-anti-human TIM-3 (#345014, BioLegend, San Diego, CA, USA), PE-anti-human LAG-3 (#369306, BioLegend), PE/Cy7-anti-human CCR7 (#353226, BioLegend), FITC-anti-human CD45RA (#304106, BioLegend, San Diego, CA, USA), PE-anti-human CD69 (#310906, BioLegend, San Diego, CA, USA), APC-anti-human CD3 (#300312, BioLegend, San Diego, CA, USA), FITC-anti-human Eomes (#11-4877-41, eBioscience, San Diego, CA, USA), PE-anti-human P-Zap70 (#12-9006-4, eBioscience), PE-anti-human granzyme B (GzmB) (#MHGB04, Invitrogen) and PE-anti-human T-bet (#12-5825-82, eBioscience, San Diego, CA, USA). Isotype-matched, nonreactive fluorescently-labeled mAbs were always used as a fluorescence reference. LSRFortessa flow cytometer (BD Biosciences, San Jose, CA, USA) was used to acquire the cells and results were analyzed in FlowJo software (Tree Star Inc., San Carlos, CA, USA).

### 4.8. LDH Release Assay

The cytotoxicity of iKP-19 CAR-T cells or CD19-CAR-T cells against Daudi cells or normal B cells was evaluated by using a standard lactic dehydrogenase (LDH) release assay (Promega, Madison, WI, USA) as described earlier by Song et al. [50]. Briefly, target cells were seeded at a density of 10^4^ cells per well in 96 well plate in a triplicate manner. The infection rate of the two CAR-T cells was adjusted to be similar, and an equal volume of effector cells and medium were added in order to make a different E:T ratio. After that 50 μL of each sample was transferred to plate in order to measure absorbance via plate reader (Thermo Fisher Scientific, Waltham, MA, USA). Results were calculated by using formulas provided by Promega.

### 4.9. Cytokine Assay

Cytokines such as IL-6, TNF-α and IFN-γ in supernatants collected from iKP-19-CAR-T cells and CD19-CAR-T cells against Daudi cells at a 1:1 ratio for 6 h or in sera from mice at day 25 were determined using a Cytometric Bead Array (CBA) assay kit (BD) according to the manufacturer’s instruction.

### 4.10. In Vivo Daudi-Derived Xenograft Model

Six- to eight-week-old NOD-Prkdc^em26Cd52^IL2rg^em26Cd22^/Nju (NCG) mice (GemPharmatech, Nangjing, China) were bred and housed under pathogen-free conditions in the animal experiment facility of East China Normal University. Mice (*n* = 4/group) were I.V. injected with 1 × 10^6^ Daudi-luc cells at day 0, followed by 5 × 10^6^ normal B cells at day 6. At day 7, Mice were treated with 5 × 10^6^ UT, CD19-CAR-T or iKP-19 CAR-T. Tumor burden was evaluated by bioluminescence (BLI) using Xenogen IVIS Imaging System with Living Image software (Xenogen Biosciences, Cranbury, NJ, USA). 150 mg/kg of D-luciferin (#115144-35-9, Merck, NJ, USA) was administered intraperitoneally to examine the tumor burden at specified time points. At day 25, Peripheral Blood (PB) of the mice was obtained from the eyelids. Daudi cell (CD19^+^HLA-C1^−^) and normal B cell (CD19^+^HLA-C1^+^) survival rate was analyzed by using flow cytometry, and cytokines release was determined by using CBA assay kits. The mice were euthanized at day 32, T cells (CD3^+^) persistence, T_CM_ (CCR7^+^CD45RA^−^) percentage, and exhaustion marker expression were analyzed by using flow cytometry. All procedures were performed in compliance with the institutional animal care committee of East China Normal University (m20190315).

### 4.11. Statistical Analysis

Statistical analysis was performed by using GraphPad prism software version 6 (La, Jolla, CA, USA). All of the in vitro experiments were performed in triplicate and the in vivo xenograft model contained 4 mice in each group. The data was analyzed by using a unpaired 2-tailed Student t test and the overall survival (OS) rate of the mice was determined by using a Mantle-Cox test. *p* < 0.05 was considered as statistically significant. The data is presented as mean ± SD.

## 5. Conclusions

The “on-target off-tumor” effect is a serious barrier to the clinical application of CAR-T cells. If CD19-CAR-T cells clear all healthy B cells, this will cause an infection in patients. We developed a KIR/PD-1-based inhibitory CAR (iKP CAR) and demonstrated that CD19-CAR-T cells bearing an iKP CAR could control B cell malignance effectively but spare healthy B cells both in vitro and in vivo. Furthermore, iKP-19-CAR-T cells exhibited a more naïve, less exhausted phenotypes and preserved a higher proportion of central memory T cells (T_CM_). Our data support that iKP CAR can be developed into a clinically implementable and promising strategy to overcome “on-target off-tumor” toxicity.

## Supplementary Materials

The following are available online at https://www.mdpi.com/2072-6694/12/9/2612/s1, Figure S1: HLA-C1 expression in primary T cells; Figure S2: The expression of P-Zap70 in CD19-CAR-T or iKP-19-CAR-T cells at day 10.
