# Supplementary material for: CD19-CAR-T Cells Bearing a KIR/PD-1-Based Inhibitory CAR Eradicate CD19+HLA-C1− Malignant B Cells While Sparing CD19+HLA-C1+ Healthy B Cells
Source: Cancers (Basel). 2020 Sep 13;12(9):2612. doi: 10.3390/cancers12092612 (PMC7564565; doi:10.3390/cancers12092612)
Supplement: Supplementary file 1 [file cancers-12-02612-s001.pdf]

# Supplementary Materials: CD19-CAR-T Cells Bearing a KIR/PD-1-Based Inhibitory CAR Eradicate CD19⁺HLA-C1⁻ Malignant B Cells While Sparing CD19⁺HLA-C1⁺ Healthy B Cells †

**Lei Tao, Muhammad Asad Farooq, Yaoxin Gao, Li Zhang, Congyi Niu, Iqra Ajmal, Ying Zhou, Cong He, Guixia Zhao, Jie Yao, Mingyao Liu and Wenzheng Jiang**

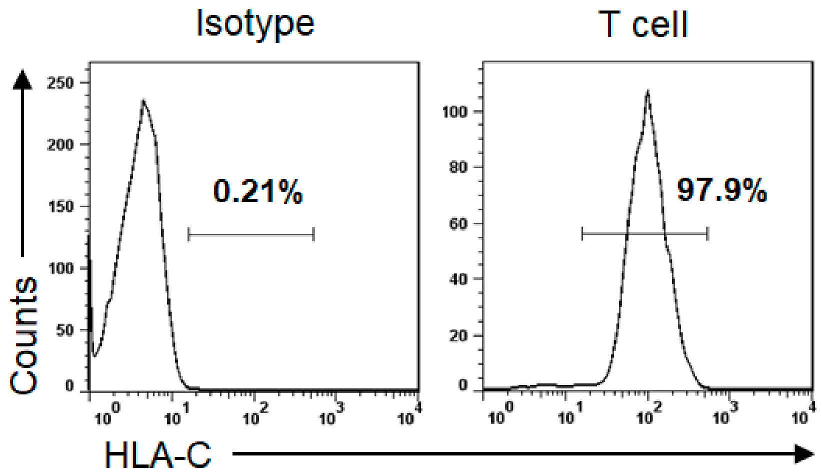


**Figure S1.** HLA-C1 expression in primary T cells. HLA-C1 expression on surface of T cells was analyzed by flow cytometry with PE-anti-human HLA-C antibody. Data are representative of 3 different donors.

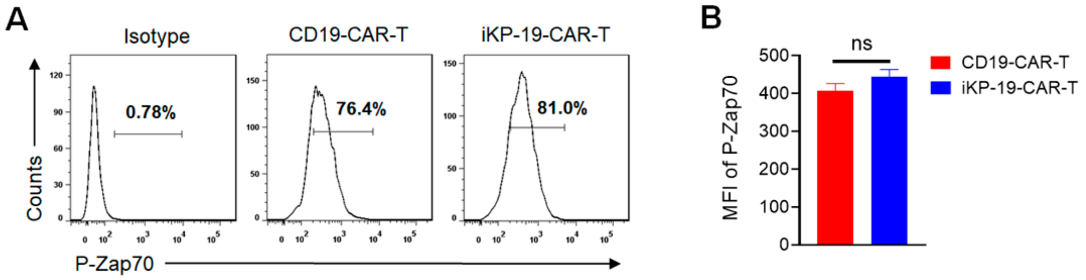


**Figure S2.** The expression of P-Zap70 in CD19-CAR-T or iKP-19-CAR-T cells at day 10. **A,** The expression of P-Zap70 in CD19-CAR-T or iKP-19-CAR-T cells was assayed by flow cytometry with PE-anti-human P-Zap70 antibody and data are representative of 3 different experiments. **B**, MFI of P-Zap70 was statistically analyzed. ns, not significant. Error bars represent ± SD.

© 2020 by the authors. Licensee MDPI, Basel, Switzerland. This article is an open access article distributed under the terms and conditions of the Creative Commons Attribution (CC BY) license (http://creativecommons.org/licenses/by/4.0/).
